# Sustainable extraction of bioactive compound from apple pomace through lactic acid bacteria (LAB) fermentation

**DOI:** 10.1038/s41598-023-46584-0

**Published:** 2023-11-07

**Authors:** Monika Kalinowska, Ewelina Gołebiewska, Małgorzata Zawadzka, Renata Choińska, Kamila Koronkiewicz, Katarzyna Piasecka-Jóźwiak, Marzena Bujak

**Affiliations:** 1 Department of Chemistry, Biology and Biotechnology, Faculty of Civil Engineering and Environmental Science, Institute of Civil Engineering and Energetics, Bialystok University of Technology, Wiejska 45E Street, 15-351 Bialystok, Poland; 2 Department of Fermentation Technology, Prof. Waclaw Dabrowski Institute of Agricultural and Food Biotechnology–State Research Institute (IBPRS-PIB), Rakowiecka 36 Street, 02-532 Warsaw, Poland

**Keywords:** Natural products, Biotechnology, Ecology, Environmental sciences, Chemistry

## Abstract

Apple pomace (AP), a by-product of the juice industry, is a rich and inexpensive source of natural bioactive substances, including phenolic compounds, that exhibit health–promoting effects. The recovery of these compounds from plant material using only classical extraction techniques and environmentally friendly solvents is often ineffective due to the entrapment of some compounds in the complex structures of plant cell walls. Lactic Acid Bacteria (LAB) fermentation can be a simple technology to increase the content of phenolic compounds, as well as the antioxidant activity of plant material. In this study, pomace from conventionally grown apples (*Malus Domestica*) of the Ligol cultivar were fermented with selected LAB strains (*Lpb. plantarum* KKP 3182, *Lpb. plantarum* KKP 1527, *Lpb. plantarum* ZFB 200), commercial starter cultures of *Lpb. plantarum*, and spontaneously. The fermented material was then subjected to ultrasound-assisted extraction, and the resulting extracts were analysed for their composition (phenolic compounds, triterpenoids, simple organic acids), and antioxidant activity. We found that: (1) the total phenolic content of AP extracts fermented with *Lpb. plantarum* KKP 1527 was about 30% higher than that of non-fermented AP extracts, (2) extracts of AP fermented with *Lpb. plantarum* KKP 1527 characterized a higher value of the antioxidant activity, (3) an increase in gallic acid procyanidin A2, protocatechuic acid, and procyanidin B2, while a decrease in rutin and quercetin was observed. The results indicated that AP fermented with *Lpb. plantarum* KKP 1527 may be a powerful and low–cost source of natural antioxidants which have applications in many industries.

## Introduction

The increasing demand for bioactive compounds has intensified research into developing the most efficient methods of obtaining them. Poland, as one of the major apple producers in Europe and worldwide, has a well-developed apple-processing sector, which generates significant amounts of by-products (about 0.5 million tons of AP/year)^1^. AP is generally composed of peels, pulp, leftover flesh, and a core with seeds and stems^2^. Due to their high water (66.4–78.2% d.w. AP) and sugar content (48.0-62.0% d.w. AP), fresh AP are easily perishable (biologically unstable), posing a significant problem for the processing industry and the environment^1,3^. As reported in the literature, apples (*Malu*s spp.) are a valuable source of dietary phytochemicals such as phenolic compounds, the content of which varies depending on the apple variety and the way the apple is cultivated, stored, and processed^4,5^. There are many reports in the literature confirming the positive effects of apple consumption on human health^6–12^.

In line with the idea of sustainable development and the circular economy, there is an emerging opportunity to reuse waste from the agri-food industry, which is a rich source of various active substances. AP can be successfully used to extract valuable phenolic compounds, which has positive ecological and economic aspects^13^. However, in many cases, a significant amount of these compounds are not reused due to entrapment in the complex structures of plant cell walls^14,15^. Fermentation using LAB can effectively influence the release of these substances, allowing extracts of high quality and bioactive compounds content to be obtained using environmentally friendly and economically viable techniques^16^. LAB are a group of Gram-positive bacteria that are commonly used in the food industry for fermentation and preservation of food and beverages (e.g. yoghurt, cheese, pickled vegetables, vinegar, wine). During the fermentation process, LAB produces anti-microbial metabolites, such as lactic acid (the major product of glucose fermentation), acetic acid, and propionic acid^17^. The process takes place under microaerophilic or strictly anaerobic conditions and involves the processing of sugars. LAB are catalase-negative, non-spore-forming, have no cytochromes, and because of their metabolism tolerate low pH^18^. The core group of LAB consists of four genera: *Lactobacillus*, *Leuconostoc*, *Pediococcus*, and *Lactococcus*. However, recent taxonomic revisions have proposed several new genera which include: *Aerococcus*, *Alloiococcus*, *Carnobacterium*, *Dolosigranulum*, *Enterococcus*, *Globicatella*, *Lactococcus*, *Oenococcus*, *Tetragenococcus*, *Vagococcus*, and *Weissella*^19^.

Antioxidant extraction using LAB has been evaluated in a number of published studies. Several papers report an increase in phenolic compounds content and antioxidant activity when LAB was used either to improve the phenolic profile of plant extracts (Table 1). In a study by Kuria et al.^20^, two LAB strains: *Lpb. plantarum* and *Lcb. casei* were used for mango pulp fermentation. Fermentation with *Lpb. plantarum* strain resulted in a greater increase in TPC (3.45 ± 0.01 mg TAE (tannic acid equivalents) g/100g of fresh weight) than fermentation with *Lcb. casei* (2.47 ± 0.01 mg TAE g/100g of fresh weight)^20^. In another study, subjecting soybeans^21^ to fermentation with *Lpb. plantarum*, *Lb. delbrueckii*, *B. breve*, and *B. thermophilum* resulted in a significant increase in antioxidant capacity and daidzein and genistein (isoflavone aglycones) content compared to the control sample. The highest increase in daidzein concentration was observed for samples fermented with *B. breve* (6 times higher), while the genistein concentration was the highest for samples fermented with *B. thermophilum* (10 times higher)^21^.

**Table 1:** Examples of LAB used in the fermentation of waste from the agro-food industry.

Plant material	LAB	Fermentation parameters	Results	References
Mango pulp*	*Lactiplantibacillus plantarum*	Temp.: 37 °C	Increase in total phenolic compounds content	^20^
(Contracted vender in Wakulima market Nakuru, Kenya)		Time: 24, 48 and 72h		
Soybean*	*Lactiplantibacillus plantarum, Lactobacillus delbrueckii, Bifidobacteria thermophilum, Bifidobacteria breve*	Temp.: 37 °C	Increase in isoflavone aglycones (genistein and daidzein) and antioxidant activity	^21^
(Pyungchang, Kangwon-do, Korea)		Time: 48 h		
Apple pomace*	*Lacticaseibacillus rhamnosus*	Temp.: 37 °C	Significant increase in total phenolic content, antioxidant activity (DPPH), gallic acid (× 20) and quercetin (× 12)	^26^
(Local fruit supermarket, Harbin, China)		Time: 7 days		
Apple juice*	*Lactiplantibacillus plantarum*	Temp.: 25 °C	Significant increase in antioxidant activity (DPPH + 23%, ABTS + 28%), decrease in total phenolic content (-3,74%) and total flavonoid content (-12,73%)	^27^
(Local fruit market, Shanghai, China)		Time: 24 h		
Apple juice*	*Lactiplantibacillus plantarum*	Temp.: 37 °C	Significant increase in antioxidant activity (DPPH + 30% by day 8, ABTS + 17% by day 8, FRAP + 15%), SOD activity (357 U/mL by day 8, no activity in raw juice), increase in total phenolic content (+ 22,45% by day 8)	^28^
(Local fruit market, Shanghai, China)		Time: 14 days		
Apple juice*	*Lactobacillus helveticus*	Temp.: 37 °C	Significant increase in phenolics: gallic acid (+ 96,89%), epicatechin (+ 38,16%), phlorizin (+ 11,73%), increase in antioxidant activity (DPPH + 4% by 36 h)	^29^
(Local fruit market, Yangling, China)		Time: 48 h		
	*Lactobacillus acidophilus*		Increase in chlorogenic acid (+ 13,27%), increase in antioxidant activity (DPPH + 5,2%)	
Apple juice*	*Lactobacillus acidophilus*	Temp.: 37 °C	Significant increase in phenolics: gallic acid (28,57%), protocatechuic acid (+ 39,77%), increase in antioxidant activity (ABTS + 35,18%) and decrease in procyanidin B2 (-28,12%)	^30^
(Xuanwu Fruit Store, Nanjing, Jiangsu Province)	*Lacticaseibacillus casei*	Time: 24 h	Significant increase in phenolics: gallic acid (27,27%), protocatechuic acid (+ 42,69%), increase in antioxidant activity (ABTS + 48,39%) and decrease in procyanidin B2 (-27,72%)	
	*Lactiplantibacillus plantarum*		Significant increase in phenolics: gallic acid (22,73%), protocatechuic acid (+ 31,58%), increase in antioxidant activity (ABTS + 42,65%) and decrease in procyanidin B2 (-29,10%)	
Blueberry pomace*	*Lacticaseibacillus casei*	Temp.: 37 °C	Significant increase in total phenolics content and antioxidant activity (DPPH + 70%, ABTS + 75%, FRAP + 94% by day 4)	^31^
(Jilin province Pulan HighTech Co., Ltd, Changchun, China)		Time: 12 days		
Cowpeas*	*Lactiplantibacillus plantarum*	Temp.: 37 °C	Increase in p-Hydroxybenzoic acid (× 3), quercetin (not detected in raw flour), decrease in antioxidant activity (DPPH -5,83%)	^32^
(wholesale market)		Time: 48 h		
Dandelion leaves*	*Lactobacillus acidophilus*	Temp.: 37 °C	Increase in total phenolics content (+ 9,33%), total flavonoid content (14,74) and antioxidant activity (DPPH + 10%); a significant increase in caffeic acid (× 4) and ferulic acid (× 5)	^33^
(Dandelion Hill Company, Jeonju, Korea)		Time: 8 days		
Djulis*	*Lactiplantibacillus plantarum*	Temp.: 37 °C	Slight increase in total phenolics content (+ 20%) and a significant increase in antioxidant activity (ABTS + 25%)	^34^
(Pingtung, Taiwan)		Time: 48 h		
Mulberry pomace*	*Lactiplantibacillus plantarum*	Temp.: 37 °C	Increase in antioxidant activity (DPPH + 80%, ABTS × 2 and FRAP × 2 by day 3); very significant increase in cyaniding (× 47), protocatechuic acid (× 120) and quercetin (× 6)	^35^
(Bailongtan HappyFarm Shayang, Hubei Province, China)		Time: 7 days		
Rice bran*	*Lactiplantibacillus plantarum*	Temp.: 37 °C	Increase in total phenolics content (+ 22%) and antioxidant activity (DPPH + 63,98%)	^36^
(Local rice milling company, Yogyakarta, Indonesia)		Time: 24 h		

*Information on the source from which the plant material was obtained was described in the publications. The information about permission to collect the plant material was not included. The plant material was mostly bought on the local market or delivered by producers.

The change in a phenolic profile that occurs during the fermentation process is a result of cellulolytic, ligninolytic, and pectinolytic enzymes that are produced by the microorganisms during growth. In the case of the *Lpb. plantarum* strain, there is a secretion of β-glucosidase and decarboxylase^22^. β-glucosidase is the enzyme responsible for catalyzing the hydrolysis of glycosidic bonds in alkyl and aryl beta-d-glucosides to release phenolic glycone moieties^16^. In addition to the release of compounds bound in complex cell wall structures, bioconversion of phenolic compounds to other substances can also occur during fermentation. The fungus *Aspergillus niger* and the bacteria *Bacillus cereus* perform glycosylation, converting catechin to catechin 4'-β-d-fucopyranoside^23^ and quercetin to isoquercetin, respectively^24^. *Lpb. plantarum* bacteria are also capable of deglycosylating quercetin glucoside to quercetin and phloridzin to phloretin^25^.

However, there are still not many reports on the fermentation of apple pomace with LAB bacteria. Fermentation is a process that requires in-depth optimization and control of the process in terms of key parameters such as pH, temperature, and the choice of starter culture itself. Different microorganisms exhibit a variety of activities within the changes in the phenolic profile of plant material. Some offer a much more efficient decomposition of complex structures with simultaneous bioconversion of compounds, while others may result in a reduction in the total content of these substances. However, an increase in the content of individual phenolic compounds is often observed despite a reduction in the total phenolic content.

There has been a trend in recent publications/interest in optimizing the fermentation process with LAB bacteria.

**Figure 1:**
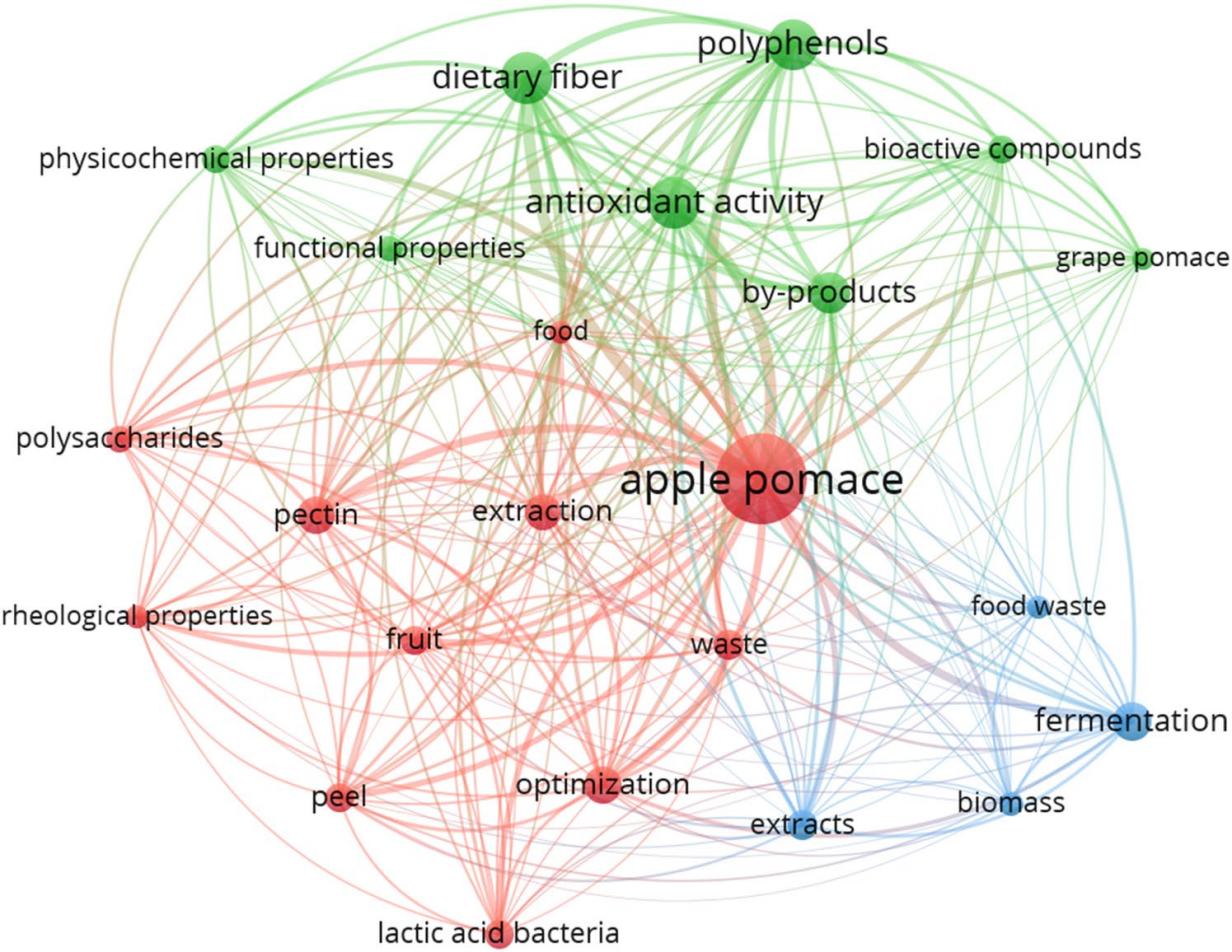
Co-occurrence of selected keywords in articles in 2014–2023 (search term: “apple pomace”), created with VOSviewer^37^.

Using the VOSviewer program, an analysis of co-occurring keywords in publications on the issue of apple pomace, from 2014 to 2023, was carried out (Fig. 1)^37^. The bibliographic data of the articles were obtained from the Web of Science database. The number 35 was set as the threshold for occurrence, resulting in 25 terms. After excluding closely related words, 23 terms remained. The program assigned them to 3 clusters. The first of these, the most numerous, consisted of terms closely related to the composition, extraction, and rheological properties of apple pomace. Among them, the pair of terms "apple pomace" and "pectin" proved to be the most intensive association, which may indicate the high interest of the authors of the publications in the extraction of these compounds. Another cluster focused on the functional properties of apple pomace and the bioactive compounds extracted from it, such as polyphenols and dietary fiber. The third, and least numerous, cluster linked the terms "fermentation," "biomass," "extracts" and "food waste," i.e., it evidenced interest in the treatment of food waste by extraction methods, such as fermentation-assisted extraction.

**Figure 2:**
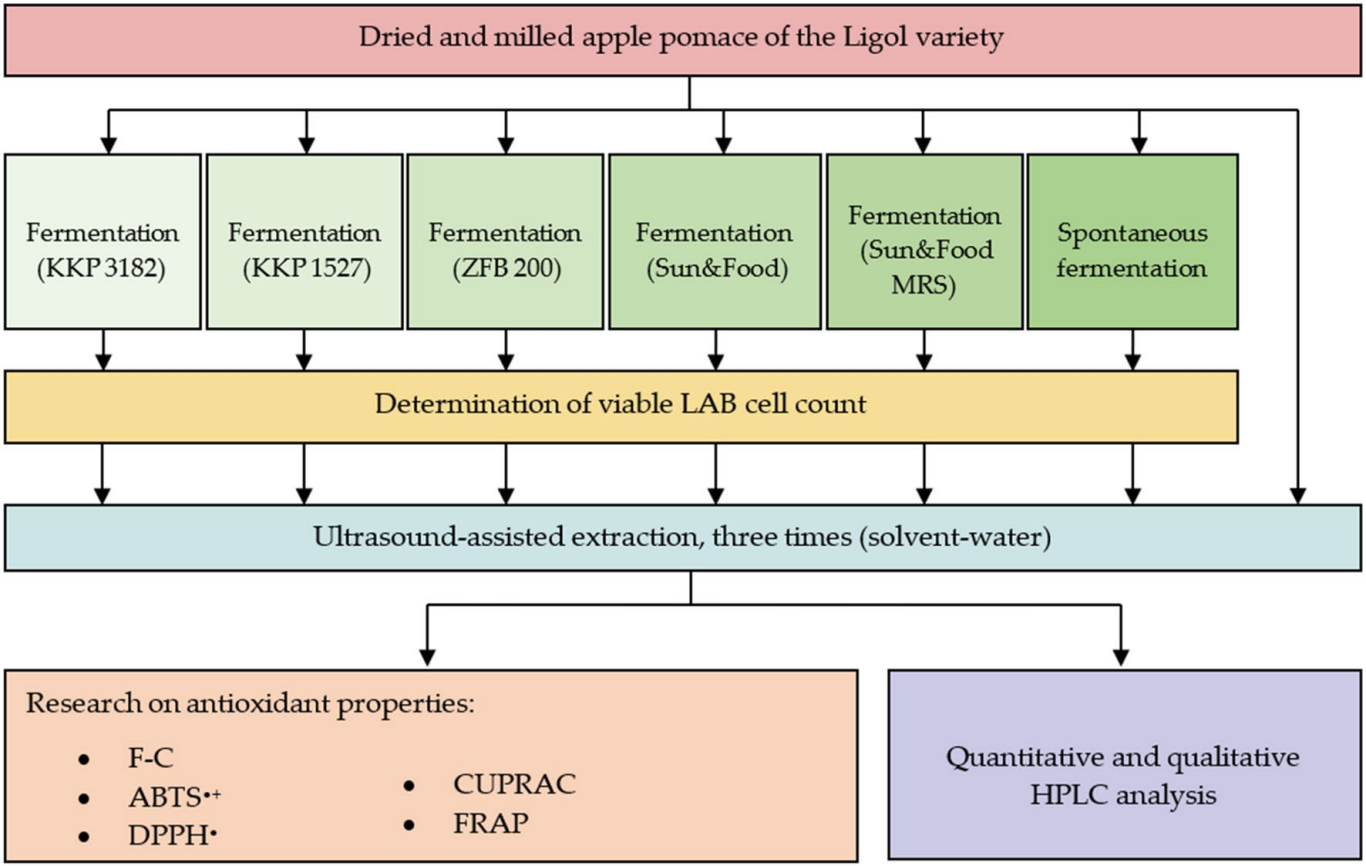
Scheme of study design.

The aim of this research was to develop an efficient method for recovering bioactive substances from AP, in line with the concept of sustainability, by optimizing the lactic fermentation process. AP fermentation was carried out using selected strains of LAB (*Lpb. plantarum* KKP 1527, *Lpb. plantarum* KKP 3182 isolated from various plant sources, and *Lpb. plantarum* ZFB 200 isolated from naturally fermented sourdough), commercial starter cultures of *Lpb. plantarum*, and spontaneous fermentation. The obtained fermented samples were analyzed for the number of viable cells and then subjected to post-fermentation treatment and UAE extraction (water was used as a solvent). Extracts were tested for total phenolic contents (Folin–Ciocalteu's method) and antioxidant activity (DPPH ((2,2-diphenyl-1-picryl-hydrazyl-hydrate)), ABTS (2,2′-azino-bis(3-ethylbenzothiazoline-6-sulfonic acid)), CUPRAC (Cupric Ion Reducing Antioxidant Capacity), and FRAP (Ferric Reducing Antioxidant Power) assays) in order to select the most optimal fermentation variant. The control sample (non-fermented AP extract) and the extracts from the fermented AP, which show the best properties, were analyzed quantitatively and qualitatively by high–performance liquid chromatography HPLC. Figure 2 shows a diagram of the research presented in this work.

## Materials and methods

### Materials

#### Plant material

Fresh apples (*Malus domestica* 'Ligol') were harvested at maturity, in 2021, from a farm located in Podlasie province in Poland. Apples were grown according to conventional methods. The fruits were harvested when ripe. The use of plant materials in the present study complies with the international, national, and institutional guidelines and legislation. The whole apples were cleaned, sliced into small pieces, and pressed in the Kuvings D9900 whole slow juicer. Then obtained apple pomaces were dried in a laboratory oven with a ventilation system (ECOCELL) at 40 °C for 24 h.

The moisture content of AP was 14% (measured with the use of MA 50/1.R Radwag moisture analyzer). The dried AP were ground using a laboratory grinder IKA A11 analytical mill.

#### Bacterial strains and culture conditions

The used strains of lactic acid bacteria i.e. *Lactiplantibacillus plantarum* KKP 1527, *Lactiplantibacillus plantarum* KKP 3182 isolated from various plant sources, and *Lactiplantibacillus plantarum* ZFB 200 isolated from naturally fermented sourdough, were obtained from the Culture Collection of Industrial Microorganisms, prof. W. Dąbrowski Institute of Agricultural and Food Biotechnology-State Research Institute in Warsaw, Poland. The strain was stored in 30% (v/v) glycerol solution in nutrient MRS broth at − 80 °C. The inoculum culture was performed in 100 mL MRS medium and incubated for 24 h at 35 °C.

Lyophilized commercial starter cultures of *Lactiplantibacillus plantarum* (EKO KISZONKI) were purchased from Sun&Food (Poland). Prior to the experiments, the starter cultures were prepared according to the manufacturer's instructions. Briefly, 0.5 g of starter culture was suspended in a sucrose solution (1.25 g of sugar dissolved in 10 mL of distilled water). In addition, for comparison purposes, the starter cultures were suspended in an MRS medium and incubated for 24 h at 35 °C.

#### Chemicals

DPPH (2,2-diphenyl-1-picrylhydrazyl), ABTS (2,2-azino-bis(3-ethylbenzothiazoline-6-sulfonic acid), potassium persulfate (K_2_S_2_O_8_), copper(II) chloride (CuCl_2_), ammonium acetate (CH_3_COONH_4_), neocuproine (2,9-dimethyl-1,10-phenanthroline), Trolox (6-hydroxy-2,5,7,8-tetramethylchroman-2–Carboxylic acid), acetate buffer, TPZT (2,4,6-tris(2-pyridil)-s-triazine), iron(III) chloride (FeCl_3_·6H_2_O), gallic acid, acetonitrile for HPLC, standards of phenolic compounds: gallic acid, protocatechuic acid, procyanidin B1, 2,5-hydroxybenzoic acid, catechin, chlorogenic acid, vanillic acid, caffeic acid, syringic acid, B2 procyanidin, (-)epicatechin, C1 procyanidin, *p*–coumaric acid, ferulic acid, rutin, procyanidin A2, quercetin-3-glucoside, quercetin, kaempferol, phloridzin, and sodium carbonate (Na_2_CO_3_) were purchased from Sigma-Aldrich Co. (St. Louis, MO, USA). Folin–Ciocalteu reagent, methanol (CH_3_OH), ethanol (C_2_H_5_OH), sodium acetate (CH_3_COONa·3H_2_O), sodium chloride (NaCl), iron(II) sulfate (FeSO_4_·7H_2_O), and sucrose (C_12_H_22_O_11_) were bought from Chempur (Piekary Slaskie, Poland). Acetic acid (CH_3_COOH) was purchased from POCH (Gliwice, Poland). Hydrochloric acid (HCl) was bought from STANLAB (Lublin, Poland). MRS medium and MRS agar were purchased from Difco (USA). All chemicals had an analytical purity and were used without further purification.

### Methods

#### Fermentation of apple pomace

**Figure 3:**
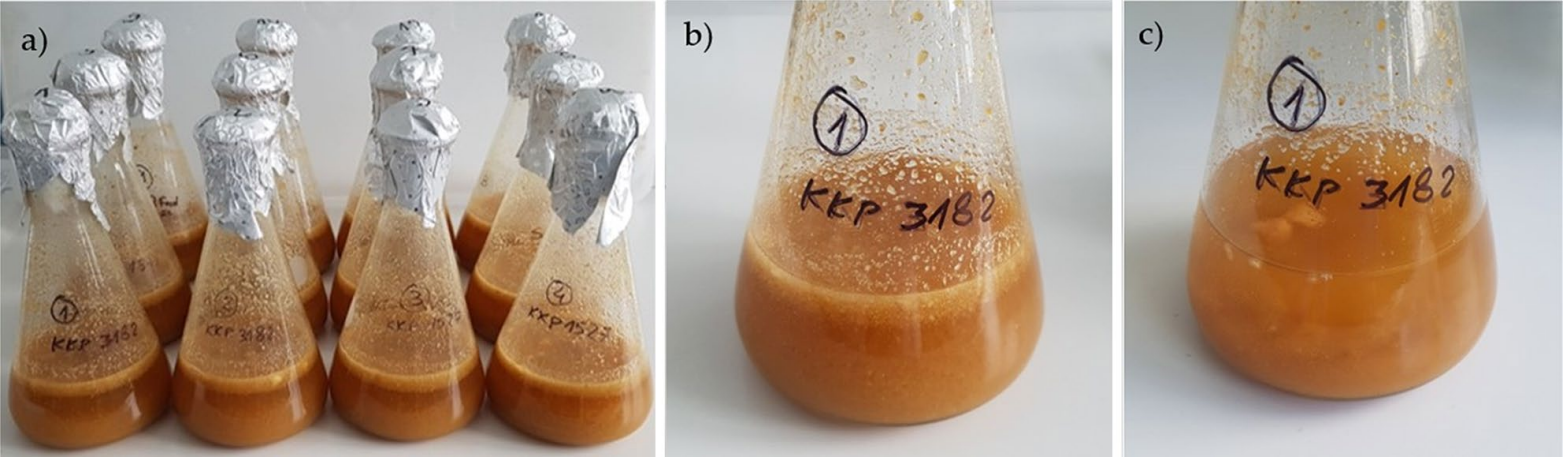
Apple pomace fermentation. (**a**) Erlenmeyer flasks before fermentation; (**b**) Erlenmeyer flask (KKP 3182) before fermentation; (**c**) Erlenmeyer flask (KKP 3182) after fermentation.

Fermentation was performed in sterilized 250 mL Erlenmeyer flasks with cotton plugs (Fig. 3). For each trial, ten grams of dried apple pomace (with an accuracy of 0.1 g) was added and poured over with 150 mL of distilled water. The samples were inoculated with 200 μL of pre-grown LAB liquid culture and commercial starter cultures. The level of LAB was 10^6^ cells/mL of apple pomace medium at the beginning of the fermentation. Fermentation was carried out for 72 h at 25 °C under aseptic conditions. Samples denoted as FS (Table 2) were fermented spontaneously i.e. without the addition of LAB strains and commercial starter cultures and served as control. The experiments were done in duplicate.

**Table 2:** Sample abbreviations used in the work.

LAC inoculum	Acronym of the sample
KKP 3182 (*Lpb. plantarum*)	KKP 3182
KKP 1527 (*Lpb. plantarum*)	KKP 1527
ZFB 200 (*Lpb.plantarum*)	ZFB 200
Sun&Food (acc. to manufacture’s instruction)	S&F
Sun&Food (MRS)	S&F MRS
Spontaneous fermentation	FS
Non-fermented	NF

#### Determination of viable LAB cell count

The viable cell count was determined in duplicate at the end of the fermentation process by plate count method on MRS agar at 35 °C for 72 h under aerobiosis. For this purpose, serial dilutions (1/10) with sterile 0.85 % saline were prepared, and 1 mL of dilution (from − 6 to − 8) was plated on MRS agar. The number of microorganisms (L), in 1 mL of the sample was calculated and the results were expressed in CFU (Colony-Forming Unit)/mL.

After the end of fermentation, the contents of the flasks were poured into beakers, dried in a water bath, and thereafter in an oven. Before further processing, the fermented pomace (the moisture content-15%) was ground again in the analytical mill.

#### Preparation of extracts

The extraction process was carried out in 250 mL borosilicate glass bottles with screw caps. 2 g of dried fermented apple pomace (with the accuracy of 0.1 g) was added to each of the 18 bottles, while 2 g of non-fermented (NF) dried apple pomace was added to the remaining three bottles (control samples). The materials were then subjected to ultrasonic extraction (in an ultrasound water bath POLSONIC SONIC-3) using 60 mL of distilled water at 25 °C for 60 minutes (ultrasound frequency 40 kHz). After cooling to room temperature, the contents of the bottles were filtered on a Büchner funnel using a vacuum pump, and the supernatant was collected. The residue was extracted twice more under the same conditions, and the supernatants were combined. The hole experiment was repeated twice.

#### Total phenolic content in extracts (Folin–Ciocalteu's spectrophotometric method)

The total phenolic content (TPC) of the fermented and non-fermented AP extracts was determined quantitatively using the Folin–Ciocalteu reagent^38^. TPC was expressed as gallic acid equivalent, i.e. mg GAE/g of extract and mg GAE/g dry weight (d.w.) of AP (equation of the standard curve in water: y= 0.0052x + 0.0226; R^2^ = 0.9981). In order to determine the standard curve, 25 mL of a 200 mg/L aqueous stock solution of gallic acid was prepared. A series of successive dilutions (170 mg/mL; 150 mg/mL; 130 mg/mL; 110 mg/mL; 90 mg/mL; 70 mg/mL; 50 mg/mL; 30 mg/mL; 25 mg/mL; 15 mg/mL) were then prepared in 10 mL flasks. To 0.25 mL of each gallic acid solution, 0.25 mL of Folin–Ciocalteu reagent diluted 1:1 by volume with deionized water, 0.5 mL of 14 % sodium carbonate solution and 4 mL of deionized water were added. After incubation in dark and at room temperature for 1 h, the absorbance of the reaction mixtures was measured at λ = 760 nm against blank sample (deionized water), using an UV/VIS/NIR Agilent Carry 5000 spectrophotometer (Santa Clara, CA, USA). To determine the TPC in AP extracts, reaction mixtures were prepared as above with one difference. Instead of successive concentrations of gallic acid, 0.25 mL of the extracts were added. The TPC of the extracts was calculated according to the formula:

1 $$TPC=\frac{{c}_{sc}\cdot {v}_{s}}{m}$$

where: TPC—total phenolic content [mg/g d.w.]; c_sc_—concentration of phenolic compounds calculated from the standard curve equation [mg/L]; v_s_—volume of solvent used for extraction [L]; m- weight of dried sample [g].

### Antioxidant activity of the extracts

#### *DPPH*^*•*^* radical scavenging assay*

A 50 µM methanolic solution of DPPH^•^ was prepared and then diluted to obtain an absorbance of ~1.200 at 516 nm. Then 2 mL of diluted DPPH^•^ solution was applied to each test tube containing 1 mL of diluted extract (150 µL extract + 850 µL of distilled water); the control contained distilled water instead of the extract. After 1 h of incubation in dark at room temperature, absorbance of each test tube was taken at 516 nm against blank sample (deionized water). The percentage inhibition (%I) of DPPH^•^ free radicals was calculated using the following equation:

2 $$\%I=\left(\frac{{A}_{control}-{A}_{sample}}{{A}_{control}}\right)\cdot 100\%$$

where: A_control_—absorbance of the control; A_sample_—absorbance of the sample.

#### *ABTS*^*•*+^*cation radical scavenging assay*

The assay was performed according to the method described by Re et al^39^. ABTS^•+^ cation radicals were produced by the reaction between aqueous solutions of 5.4 mM ABTS and 1.54 mM potassium persulfate (in a volume ratio of 1:1), stored in the dark at room temperature for 24 h before use. ABTS^•+^ solution was then diluted with distilled water to obtain an absorbance of 0.700–0.800 at 734 nm. After the addition of 100 µL of extract to 2.5 mL of diluted ABTS^•+^ solution absorbance was measured at 7 min after the initial mixing; the control contained distilled water instead of the extract. The percentage inhibition (%I) of ABTS^•+^ cation radicals was calculated according to the Eq. (2).

#### Cupric ion reducing antioxidant capacity (CUPRAC) assay

CUPRAC assay was assessed according to Apak et al^40^. Copper chloride aqueous solution (C = 10 mM), neocuproine alcoholic solution (C = 7.5 mM in absolute ethanol), and ammonium acetate buffer solution (pH 7.0) was prepared. These three reagents were mixed at a volume ratio of 1:1:1 to produce a working solution of CUPRAC. Then, 3 mL of CUPRAC working reagent was applied to each test tube containing 0.2 mL of diluted extract (100 µL extract + 100 µL of distilled water); the control contained distilled water instead of the extract. After 1h of incubation at room temperature, the absorbance of each test tube was taken at 450 nm against a blank sample (deionized water). The copper ion-reducing antioxidant activity was evaluated using the standard curve obtained for Trolox (in the concentration range 50–350 µM). Standard curve equation: y= 1.7302x − 0.0042; R^2^ = 0.9988. The data were expressed as Trolox equivalents [µM/L_extract_].

#### Ferric reducing antioxidant power (FRAP) assay

FRAP assay was performed using the method described by Rice-Evans et al^41^. FRAP working reagent was prepared by mixing 300 mM acetate buffer (pH 3.6), 10 mM TPTZ (the iron-2,4,6-tris-2-picryl-s-thiazine complex), and 20 mM FeCl_3_∙6H_2_O (20 mM) in the proportion of 10:1:1. Then, 3 mL of freshly prepared working FRAP reagent was applied to each test tube containing 0.4 mL of diluted extract (100 µL extract + 300 µL of distilled water); the control contained distilled water instead of the extract. After 15 min of incubation at room temperature, absorbance of each test tube was taken at 595 nm against blank sample (deionized water). The ferric reducing antioxidant power was evaluated using the standard curve obtained for FeSO_4_ (in the concentration range 50–550 µM). Standard curve equation: y= 1.9326x − 0.0232; R^2 ^=  0.9975. The data were expressed as Fe^2+^ equivalents [μM/L_extract_].

#### Statistical analysis

All assays were performed in five replications for three independent experiments. The results were expressed as the means of the values obtained for the replications. Data were tested for normal distribution by performing the Shapiro-Wilk test. Average, and standard deviation calculations, and graphs were completed using Microsoft Excel 2019.

#### HPLC analysis

The qualitative and quantitative chromatographic analysis of phenolic compounds present in tested extracts was performed on an Agilent 1260 Infinity liquid chromatograph with a UV spectrophotometric detector, using a Zorbax Eclipse Plus C18 Analytical precolumn column (4.6 × 250 mm; 5 µm) according to Kalinowska et al^42^. Samples, at a concentration of 0.7–0.8 g per 10 mL of methanol, underwent analysis. Phenolic compounds were analyzed using acetonitrile (A) and 2% acetic acid (B) as the mobile phase, flowing at 1 mL/min. The elution followed a gradient program: 0–40 min, a linear shift from 3% to 15% A and 97% to 85% B; 40–60 min, 15–25% A and 85–75% B; 60–75 min, 25–50% A and 75–50% B; 75–80 min, 50–95% A and 50–5% B; 80–85 min, 95–3% A and 5–97% B; and 85–90 min, 3% A and 97% B. Detection was at 280, 320, and 360 nm. For triterpenoids, a mobile phase of 90% acetonitrile and 10% water was used with isocratic elution at 0.5 mL/min, a 10 μL sample, and an analytical wavelength of 280 nm. Organic acids and ascorbic acid were analyzed with a mobile phase of 50 mM H_3_PO_4_ and 10 mM NaH_2_PO_4_, isocratically eluted at 0.8 mL/min, a 10 μL sample, and analytical wavelengths of 210 and 244 nm. Compound identification relied on retention time and DAD absorbance spectra of standards, while the content was determined using a five-point calibration curve.

#### FT-IR analysis

The solid samples in the form of KBr pellets were subjected to FT-IR analysis using an Alfa Bruker spectrometer (Bremen, Germany). The spectra were obtained and examined within the frequency range of 600–4000 cm^−1^.

## Results and discussion

### Bacterial viability

Table 3 summarizes the number of viable LAB cells obtained by plate counting after incubation for 72 h. The highest increase in the number of bacteria was found in fermented apple pomace with *Lpb. plantarum* KKP 1527, 3.8·10^8^ CFU/mL (sample KKP 1527–I and 3.6·10^8^ CFU/mL (sample KKP 1527–II). In the case of the samples treated with the other *Lpb. plantarum* strain used, i.e. *Lpb. plantarum* KKP 3182, a weaker growth (2.9·10^8^ CFU/mL) was observed. The smallest number of bacteria was obtained for samples fermented with *Lpb. plantarum* ZFB 200 (2.1·10^8^ CFU/mL), followed by S&F–I (1.8·10^7^ CFU/mL) fermented with the addition of a commercial starter culture prepared in accordance with the manufacturer's instructions. Interestingly, in the case of the commercial starter culture prepared in MRS, no single LAB colony at a dilution of 10^−6^ was found on the plate. The same was observed for spontaneous fermentation, which would indicate growth inhibition. The obtained results suggested that the commercial starter culture was not capable of efficiently growing and controlling the fermentation process in the apple pomace medium. On the other hand, the used LAB strains, isolated from plant resources showed better adaptation to the apple pomace environment, which is consistent with previous observations that the use of native LAB strains during the fermentation process is advantageous^43^.

**Table 3:** The number of viable LAB after apple pomace fermentation for 72 h at 25 °C.

Sample	Number of LAB [CFU/mL] × 10^8^
KKP 3182–I	2.71 ± 0.2
KKP 3182–II	2.88 ± 0.2
KKP 1527–I	3.76 ± 0.1
KKP 1527–II	3.62 ± 0.1
ZFB 200–I	2.15 ± 0.2
ZFB 200–II	2.07 ± 0.1
S&F–I	0.182 ± 0.2
S&F–II	No growth**
S&F MRS–I	No growth**
S&F MRS–II	No growth**
FS–I	No growth**
FS–II	No growth**

**—no single colony was found on the plate at dilution 10^−6^; I and II—no. of experiment.

### Total phenolic content in extracts

**Figure 4:**
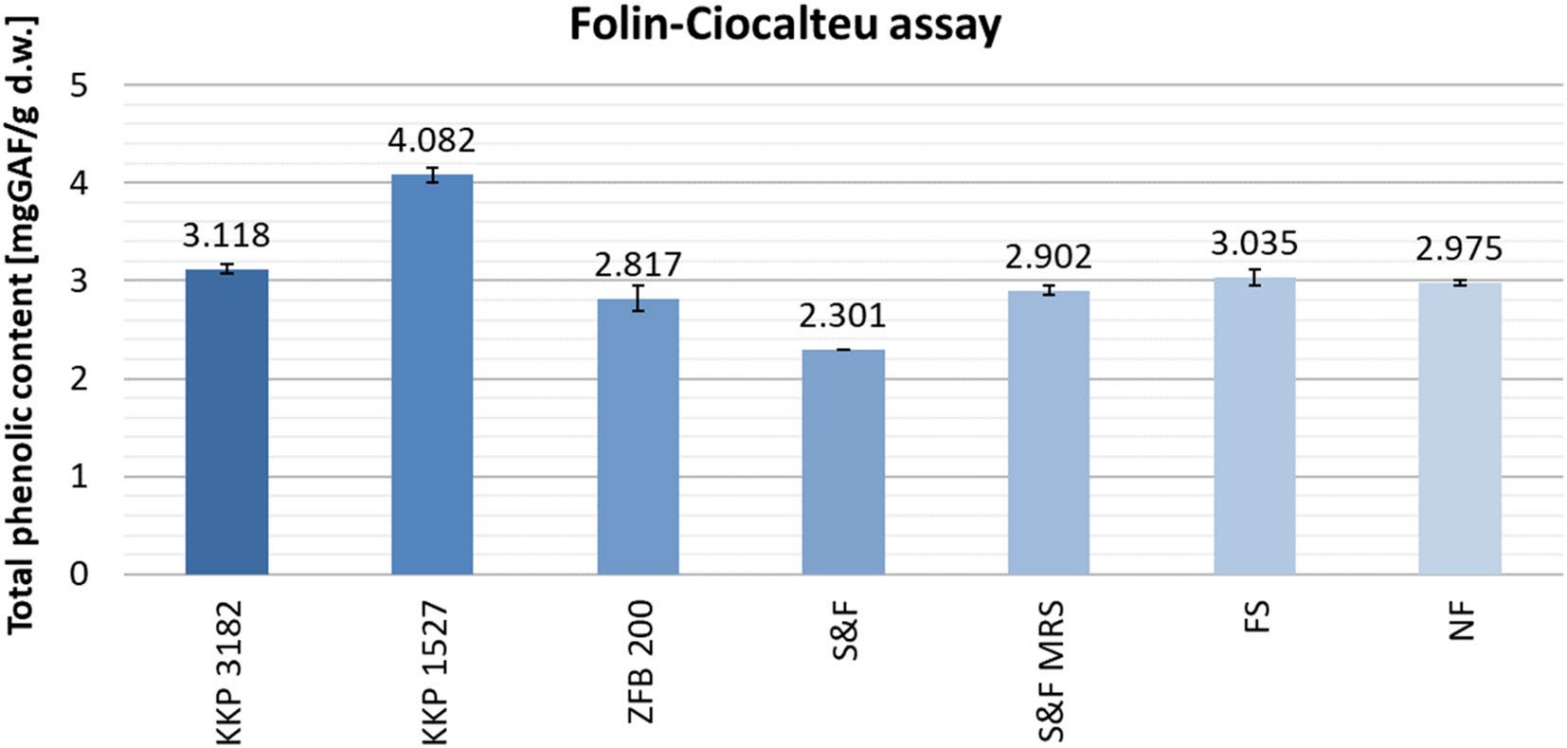
Total phenolic compounds in AP extracts, depending on pretreatment (fermentation/no fermentation) and microorganism. Mean values from three independent experiments ± SD are shown.

Figure 4 shows the total content of phenolic compounds in tested fermented and non-fermented AP extracts, expressed as mg gallic acid equivalents per g dry weight of AP (mg GAE/g d.w. AP). The highest TPC value was determined in AP fermented with *Lpb. plantarum* KKP 1527 (4.082 ± 0.076 mg GAE/g d.w. AP). Averaging the results obtained for non-fermented AP extracts yielded a value of 2.975 ± 0.031 mg GAE/g d.w. AP. Thus, samples with the highest TPC content had an increase in total polyphenol content of 1.107 mg GAE/g d.w. AP (sample KKP 1527) compared to the average TPC value of control samples (NF). AP fermented with *Lpb. plantarum* KKP 3182 also showed an increased TPC content (3.118 ± 0.045 mg GAE/g d.m. AP) (+0.143 mg GAE/g d.w. AP compared to the average TPC value of control samples). These results also overlap with the highest recorded abundance of LAB bacteria in these samples (Table 3). A significant increase in polyphenol content observed in AP fermentation with *Lpb. plantarum* may be attributed to the accumulation of β-glucosidase, an enzyme produced during fermentation. β-glucosidase plays a crucial role in hydrolyzing complex polyphenols, leading to the release of more bioactive phenolic compounds, thus contributing to the enhanced polyphenol content. This enzymatic activity enhances the potential health benefits associated with polyphenols^44^.

The largest decrease in TPC, by 0.675 mg GAE/g d.w. AP, was observed in sample S&F. The remaining AP extracts showed only a slight increase (sample FS) or slight decrease (S&F MRS, ZFB 200) in total phenolic content. In the case of the ZFB 200 sample, this may indicate the low potential of this strain to alter the phenolic profile of products or their consumption by bacteria through detoxification mechanisms. In the S&F MRS and FS samples, it is possible that fermentation did not occur.

### Antioxidant activity of extracts

Due to the complex nature of the different phytochemical classes present in plant extracts and the different mechanisms of action of the individual tests, there is no single universal method for determining the antioxidant capacity^45,46^. In the present study, we included DPPH^•^ free radical scavenging, ABTS^•+^ cation radical scavenging, cupric ion reducing antioxidant capacity, and ferric reducing antioxidant power to test the antioxidant activity of the aqueous extracts of fermented and non-fermented AP.

**Figure 5:**
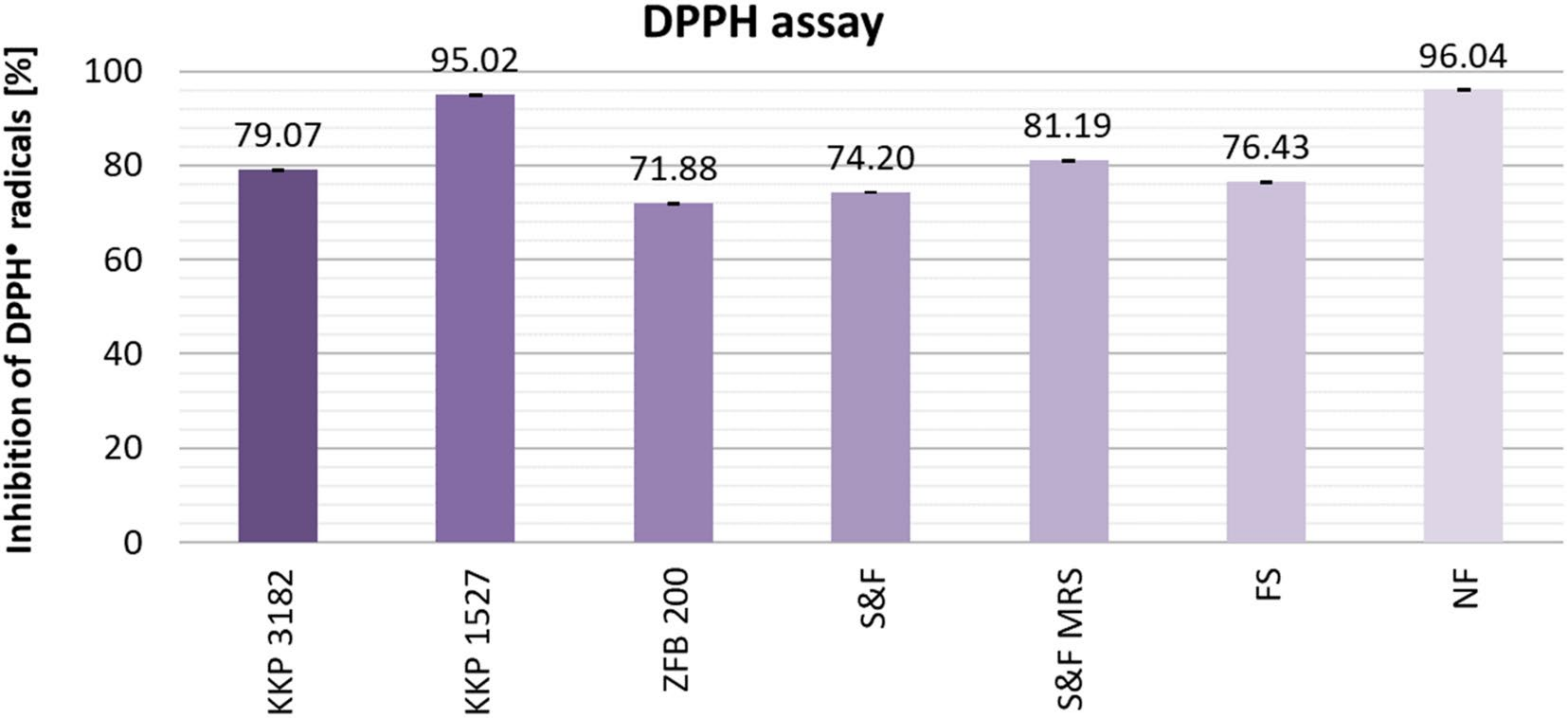
Comparison of the DPPH^•^ radical scavenging activity of AP extracts, depending on pretreatment (fermentation/no fermentation) and microorganism. Mean values from three independent experiments ± SD are shown.

Figure 5 shows the results of DPPH assay. In this test, no increase in antiradical activity was observed under AP fermentation. Non-fermented AP extracts (NF) inhibited the DPPH^•^ radicals by 96.04%. The most significant decrease in activity was observed for samples fermented with *Lpb. plantarum* ZFB 200 (− 24.16%) compared to the average %I value of control samples (NF). Samples fermented with *Lpb. plantarum* KKP 3182 (-16.97%) and Sun & Food starter cultures (S&F: − 21.84%, S&F MRS: -14.85%) also showed marked decreases. The lowest decrease in DPPH^•^ free radical scavenging activity was shown by samples fermented with *Lpb. plantarum* KKP 1527 (− 1.02%). The observed decrease in activity could be attributed in part to insufficient inoculation time during the fermentation process.

**Figure 6:**
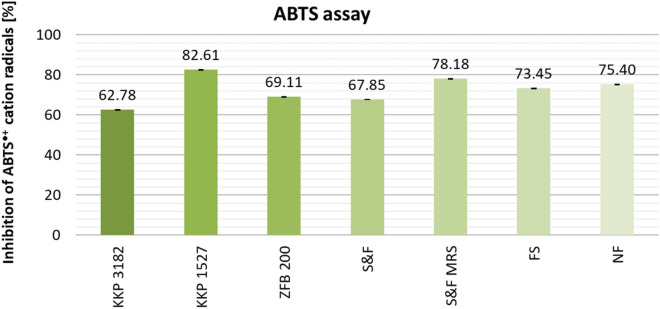
Comparison of the ABTS^•+^ cation radical scavenging activity of AP extracts, depending on pretreatment (fermentation/no fermentation) and microorganism. Mean values from three independent experiments ± SD are shown.

Figure 6 presents the results of ABTS assay. The highest ABTS^•+^ cation radical scavenging activity was observed for samples fermented with *Lpb. plantarum* KKP 1527 (%I= 82.61%). This result coincides with higher total phenolic compounds content (Figure 4) and bacterial abundance (Table 3) for KKP 1527 samples. Non-fermented AP extracts showed an ABTS^•+^ cation radical inhibition rate of 75.40%. The largest decrease in activity compared to the average activity of NF AP extracts was observed for KKP 3182 samples (− 12.62%). Samples KKP 3182 are *Lpb. plantarum* strains isolated from various plant sources, which demonstrates the importance of choosing the right source for the isolation of LAB bacteria used during fermentation. For the remaining samples (ZFB 200, S&F, FS), only slight decreases in the ability of the extracts to inhibit the ABTS^•+^ cation radicals were observed.

**Figure 7:**
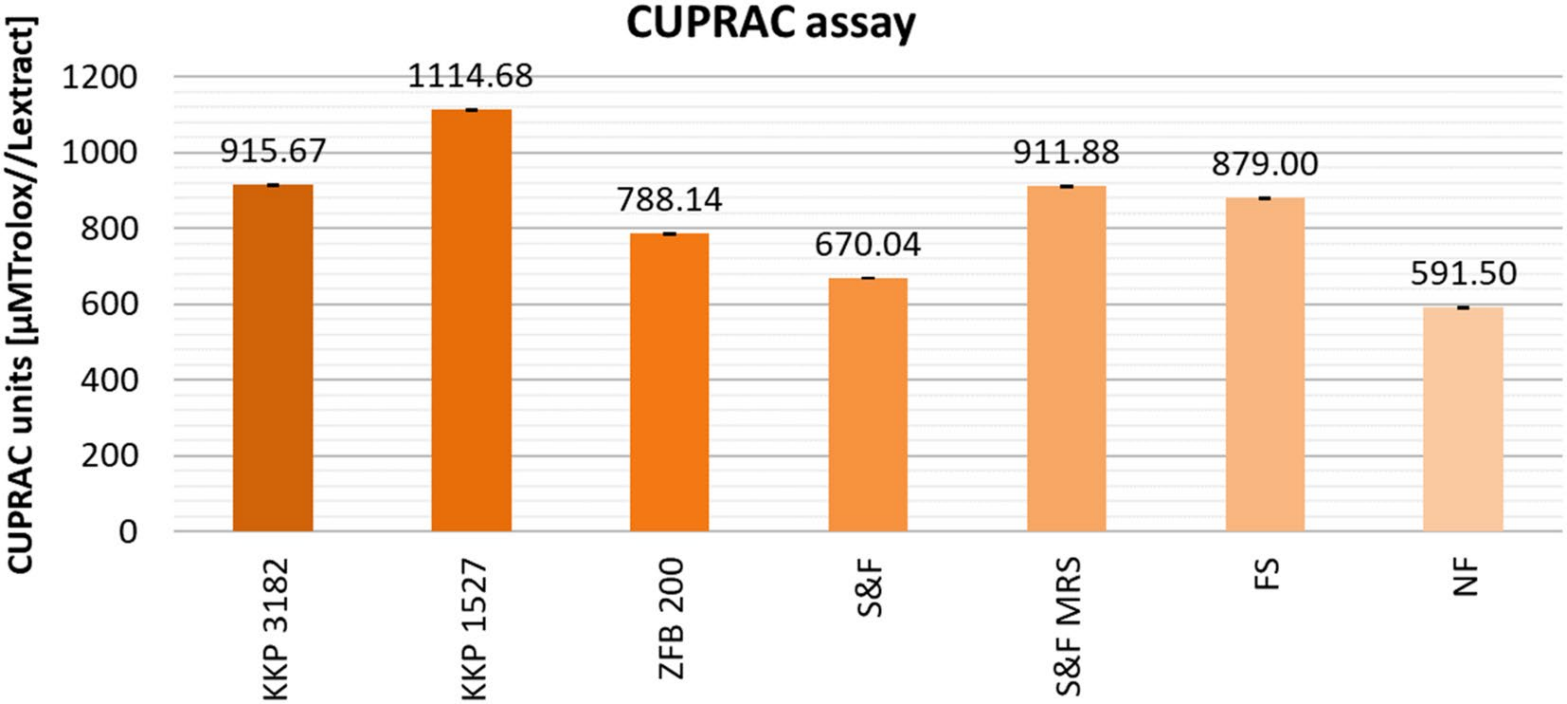
Comparison of the cupric ion reducing antioxidant capacity of AP extracts, depending on pretreatment (fermentation/no fermentation) and microorganism. Mean values from three independent experiments ± SD are shown.

The results of CUPRAC assay are illustrated in Fig. 7. Tests on the ability of extracts to reduce Cu(II) ions showed a significant increase in activity in the case of fermented samples. The CUPRAC antioxidant capacity of the tested samples ranged from 670.04 to 1114.68 µM of Trolox equivalents for fermented AP extracts and 591.50 µM of Trolox equivalents for non-fermented AP extracts. The highest increase in antioxidant properties relative to the average activity of control (NF) was observed for samples fermented with *Lpb. plantarum* KKP 1527 (+ 523.18 µM of Trolox equivalents). Samples: KKP 3182, S&F MRS, and FS showed similar reducing activity (in the range of 879.00–915.67 µM of Trolox equivalents). The lowest increase in Cu(II) ions reduction activity compared to NF AP extracts was noticed for samples S&F (+ 78.54 µM of Trolox equivalents), and ZFB 200 (196.64 µM of Trolox equivalents). Again, the dominance of the KKP 1527 (*Lpb. plantarum*) culture relative to the other variants tested was observed.

**Figure 8:**
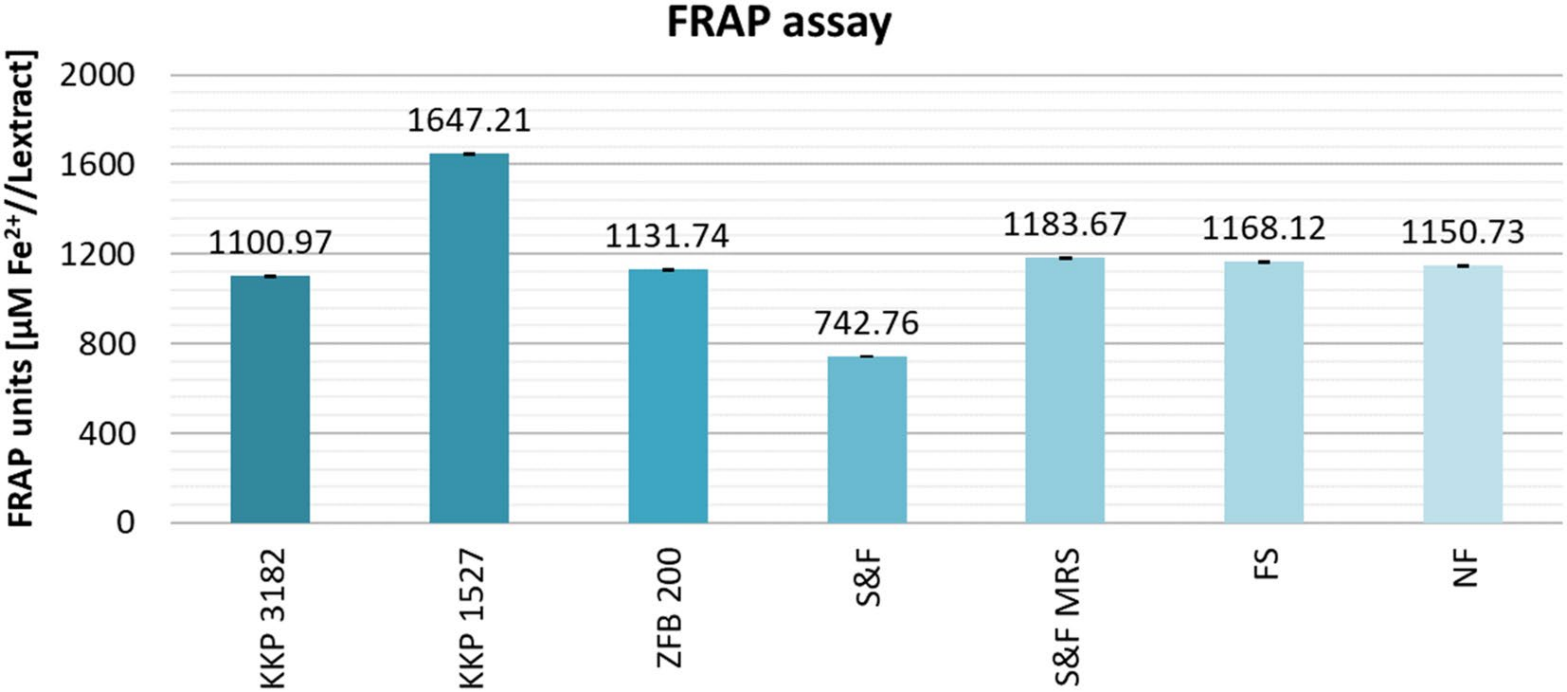
Comparison of the ferric reducing antioxidant power of AP extracts, depending on pretreatment (fermentation/no fermentation) and microorganism. Mean values from three independent experiments ± SD are shown.

Figure 8 shows the results of FRAP assay. Tests of the reducing activity of the extracts against Fe(III) ions showed a significant increase in these properties for samples fermented with *Lpb. plantarum* KKP 1527 (+496.48 µM Fe^2+^/L_extract_) compared to the average activity value of NF samples. Samples: S&F MRS, and FS showed only a slight increase in iron(III) reducing capacity. The largest decrease in reducing activity was observed for the sample S&F (− 407.97 µM Fe^2+^/L_extract_). For the remaining samples (KKP 3182, and KKP 1811), the decreases in activity were negligible.

Summarizing the results of antioxidant assays (DPPH, ABTS, CUPRAC, FRAP), aqueous extracts of AP fermented with *Lpb. plantarum* KKP 1527 (samples KKP 1527) showed the strongest antioxidant activity (Figs. 5, 6, 7 and 8). The DPPH assay showed a decrease in antioxidant activity for all extracts. However, for *Lpb. plantarum* KKP 1527 strain used, the decrease in DPPH^•^ radical inhibition compared to the NF AP extracts was the lowest (1.02%). It was observed that the highest bacterial abundance in samples fermented with *Lpb. plantarum* KKP 1527 contributed to an increase in antioxidant activity and indicated the high potential of this strain as a starter for apple pomace fermentation. During fermentation, many bacterial strains not only release bioactive compounds from the complex cell wall structures of plant materials but may also release enzymes that cause the transformation of these compounds, resulting in a decrease in their activity^14,15^. The Folin–Ciocalteu assay results for TPC (Fig. 4) also had a significant impact on the antioxidant activity results. The extracts that showed a higher content of phenolic compounds possessed higher antioxidant properties. This shows that the main changes in the content of antioxidant compounds, occurring as a result of fermentation, are related mostly to changes in the phenolic profile of apple pomace^22^. There are many studies on the effect of fermentation with LAB on increasing the phenolic compounds content and antioxidant activity of plant material, including apple pomace^26,47,48^. Liu et al.^26^, showed that fermentation with *Lacticaseibacillus rhamnosus* is a promising way to improve the bioactivity of phenolic compounds in apple pomace. However, further research is needed to thoroughly investigate the effect of LAB on phenol metabolism^26^. Tang et al.^35^, found that fermentation of mulberry pomace with *Lactiplantibacillus plantarum* had a positive effect on the phenolic compound content and antioxidant activity of the resulting extracts. This result may be related to the production of new phenolic acids and an increase in the concentration of aglycones and phenolic acids^35^.

### HPLC analysis

The extract with the highest antioxidant activity (from AP fermented with *Lpb. plantarum* KKP 1527) and the extract from non-fermented AP, as a control sample, were selected for HPLC analysis. The results showed a significant increase in the total content of phenolic compounds (TCPC) in extracts obtained from AP fermented with *Lpb. plantarum* KKP 1527 compared to extracts obtained from unfermented AP (Table 4). In the case of non-fermented AP, the value of TCPC was 1720.905 ± 40.340 µg/g d.w. of extract and 733.120 ± 11.343 µg/g d.w. of AP, while for fermented AP the TPTC value was 2281.284 ± 49.760 µg/g d.w. of extract and 1285.902 ± 28.048 µg/g d.w. of AP. Fermentation of AP with *Lpb. plantarum* KKP 1527 increased the total content of phenolic compounds by 32.6 % (+560.379 µg/g d.w. extract) and 75.4 % (+ 552.782 µg/g d.w. AP), which was also confirmed by the results of the Folin–Ciocalteu assay (Fig. 4).

**Table 4:** Comparison of the phenolic compounds composition (HPLC analysis) of non-fermented AP extracts versus AP extracts fermented with *Lpb. plantarum* (KKP 1527) [µg/g d.w. extract or AP]. Mean values from three independent experiments ± SD are shown.

Lp	Phenolic compound	λ [nm]	TR [min]	µg/g d.w. extract		µg/g d.w. AP	
				NF	KKP 1527	NF	KKP 1527
1	Gallic acid	280	7.0	18.762 ± 0.099	121.571 ± 2.633	7.922 ± 0.042	68.527 ± 1.484
2	Protocatechuic acid	280	10.6	11.145 ± 6.192	42.220 ± 0.924	4.705 ± 2.614	23.798 ± 0.521
3	Procyanidin B1	280	15.8	33.613 ± 4.724	109.716 ± 8.843	14.192 ± 1.995	61.844 ± 4.985
4	2,5-dihydroxybenzoic acid	320	17.5	–	–	–	–
5	Catechin	280	19.8	1152.744 ± 8.440	1277.079 ± 8.229	486.692 ± 3.563	719.857 ± 4.639
6	Chlorogenic acid	320	20.9	190.330 ± 11.988	207.679 ± 9.441	80.358 ± 5.061	117.064 ± 5.322
7	Vanillic acid	280	22.6	–	–	–	–
8	Caffeic acid	320	24.0	16.568 ± 1.646	11.867 ± 1.836	6.995 ± 0.695	6.689 ± 1.035
9	Syringic acid	280	26.2	–	–	–	–
10	Procyanidin B2	280	28.0	–	–	–	–
11	(–)Epicatechin	280	29.4	78.177 ± 20.698	107.403 ± 18.904	33.006 ± 8.739	60.540 ± 10.656
12	Procyanidin C1	280	32.9	–	–	–	–
13	*p*–coumaric acid	320	34.8	–	–	–	–
14	Ferulic acid	320	40.5	–	–	–	–
15	Rutin	360	46.8	57.788 ± 2.207	7.696 ± 1.085	24.398 ± 0.932	4.338 ± 0.611
16	Procyanidin A2	280	47.4	79.779 ± 4.828	326.537 ± 31.318	33.683 ± 2.038	184.060 ± 17.653
17	Quercetin-3-glucoside	360	48.1	40.655 ± 1.332	31.105 ± 0.740	17.165 ± 0.562	17.533 ± 0.417
18	Phloridzin	280	57.6	54.185 ± 5.758	29.456 ± 10.134	22.877 ± 2.431	16.604 ± 5.712
19	Quercetin	360	66.3	13.752 ± 1.229	8.954 ± 0.930	5.806 ± 0.519	5.047 ± 0.524
20	Kaempferol	360	71.3	–	–	–	–
TOTAL PHENOLICS (TPTC)				1720.905 ± 40.340	2281.284 ± 49.760	733.120 ± 11.343	1285.902 ± 28.048

The phenolic profiles of apples vary depending on apple varieties and the specific parts of the fruit, such as the pulp and peel, contributing to their unique composition^1^. Gallic acid, protocatechuic acid, procyanidin B1, catechin, chlorogenic acid, caffeic acid, (–)epicatechin, rutin, procyanidin A2, quercetin-3-glucoside, phloridzin, and quercetin were found in both AP extracts. Compounds such as 2,5-dihydroxybenzoic acid, vanillic acid, syringic acid, procyanidins B2 and C1, *p*–coumaric acid, ferulic acid, and kaempferol were not detected. The extracts obtained from non-fermented AP were abundant in catechin, chlorogenic acid, (–)epicatechin, procyanidin A2, rutin, and phloridzin, while fermented AP extracts contained the most catechin, procyanidin A2, chlorogenic acid, gallic acid, procyanidin B1, and (–)epicatechin. Although the fermented AP extract possessed higher total content of phenolic compounds, some of the individual phenolics were more abundant in the non-fermented AP extract, especially rutin accounting for 24.398 µg/g d.w. AP to 57.788 µg/g d.w. extract, and phloridzin (22.877 µg/g d.w. AP to 54.185 µg/g d.w. extract).

Based on the observations of the concentration of individual compounds, a significant change in the phenolic profile of the samples under fermentation with the *Lpb. plantarum* KKP 1527 was found. The main increase was noticed for gallic acid, procyanidin A2, protocatechuic acid, and procyanidin B2. The contents of gallic acid, and procyanidin A2 in the extracts were approximately 6.5, and 4.1 times higher in samples fermented with *Lpb. plantarum* KKP 1527 compared to the corresponding phenolic acids in the control samples. In addition, a significant decrease in rutin (by 86.7%) and phloridzin (by 45.6%) content was observed in the fermented extracts relative to the non-fermented extracts.

The content of selected terpenoids and organic acids (including ascorbic acid) in the tested samples is shown in Table 5. Only betulinic acid was detected in non-fermented AP. Betulinic acid, tartaric acid, and malic acid were detected in the AP fermented with *Lpb. plantarum* KKP 1527. The content of betulinic acid in the fermented samples (average values: 362.891 µg/g d.w. of extract and 148.310 µg/g d.w. of AP) was higher than in the non-fermented samples (average values: 351.275 µg/g d.w. of extract and 204.553 µg/g d.w. of AP).

**Table 5:** Comparison of the triterpenoids and organic acids (including ascorbic acid) composition (HPLC analysis) of non-fermented AP extracts versus AP extracts fermented with *Lpb. plantarum* (KKP 1527) [µg/g d.w. extract or AP].

Lp		µg/g d.w. extract		µg/g d.w. AP	
		NF	KKP 1527	NF	KKP 1527
Triterpenoids					
1	Betulinic acid	351.275 ± 41.224	362.891 ± 13.205	148.310 ± 17.405	204.553 ± 7.443
2	Oleanolic acid	–	–	–	–
3	Ursolic acid	–	–	–	–
Organic acids					
1	Tartaric acid	–	538.574 ± 82.432	–	303.580 ± 46.465
2	Malic acid	–	13,695.633 ± 322.168	–	7719.884 ± 181.598
3	Citric acid	–	–	–	–
4	Quinic acid	–	–	–	–
5	Ascorbic acid	–	–	–	–

Mean values from three independent experiments ± SD are shown.

During the process of fermentation, microorganisms play a crucial role in synthesizing enzymes that possess the unique ability to break ester bonds. This enzymatic action results in the release of phenolic acids that were previously bound within the plant material. The improvement of phenolic contents in plants is often linked to the activity of various enzymes produced by microorganisms, including notable examples such as β-glucosidase, α-amylase, and laccase, along with other enzymes. These enzymes work collectively to facilitate the breakdown of complex phenolic compounds, leading to an increase in the availability of individual phenolic monomers^36,49^. However, typically, differences in enzyme production can be attributed to specific strain characteristics, such as genetic composition and metabolic capacity, as well as environmental and nutritional conditions that affect their growth^50^.

A significant increase in gallic acid levels observed during AP fermentation with *Lpb. plantarum* KKP 1527 in our work can be attributed to the enzymatic activities of *Lpb. plantarum*, specifically the action of esterases, glycosidases, and tannases. This pathway enables the hydrolysis of tannic acid, resulting in the formation of gallic acid and glucose. Following this, gallic acid undergoes decarboxylation, leading to the production of pyrogallol. The enzymatic reactions responsible for this metabolic transformation are tannase and gallate decarboxylase, which act successively to catalyze the hydrolysis of tannic acid and the subsequent decarboxylation of gallic acid. Through the activity of these enzymes, *Lpb. plantarum* facilitates the conversion of tannic acid into gallic acid and pyrogallol, contributing to the overall increase in gallic acid levels during fermentation^51–53^. The observed significant decrease in phloridzin content during AP fermentation with *Lpb. plantarum* KKP 1527 indicates the potential conversion of phloridzin to phloretin. This conversion is likely facilitated by the enzymatic hydrolysis of phenolic compounds, including flavonoid glycosides, which results in the formation of their corresponding aglycones. However, the definitive determination of this conversion is limited by the absence of a higH–performance liquid chromatography (HPLC) standard for phloretin. Further studies with appropriate standards are necessary to confirm the transformation of phloridzin to phloretin and elucidate the precise mechanism underlying this enzymatic conversion during LAB fermentation of AP^26^. The enzyme rutinase plays a crucial role in the hydrolysis of rutin into its aglycone form, quercetin. In our study, we observed a substantial decrease in rutin content following fermentation, with a reduction of 86.7%. Surprisingly, there was no corresponding increase in the content of quercetin and quercetin-3-glucoside; instead, we observed a decrease in their levels. This finding suggests that the conversion of rutin by the fermentation process did not result in the accumulation of quercetin or its glucoside derivatives. The conversion of rutin by LAB can vary depending on the LAB strain, fermentation conditions, and substrate composition. Factors such as the presence of other phenolic compounds and nutrients in the fermentation matrix can influence enzymatic activities and subsequent conversion processes. Understanding this conversion is essential for producing metabolites with potential health benefits and developing fermented foods with improved phenolic profiles. However, further research is needed to uncover the specific enzymes, reactions, and bioactive properties associated with the conversion of rutin by different LAB strains^54,55^.

Overall, the fermentation process acts as a transformative mechanism that effectively cleaves the bonds between phenolic compounds and other substances. This enzymatic activity liberates the monomers of phenolic compounds or antioxidants, thereby enhancing the overall phenolic content and antioxidant activity of the fermented product. Such transformations during fermentation have significant implications for, among other things, the development of novel food and beverage products with enhanced nutritional and health–promoting properties, as well as the efficient recovery of bioactive substances from agri-food industry waste^36^.

### FT-IR analysis

**Figure 9:**
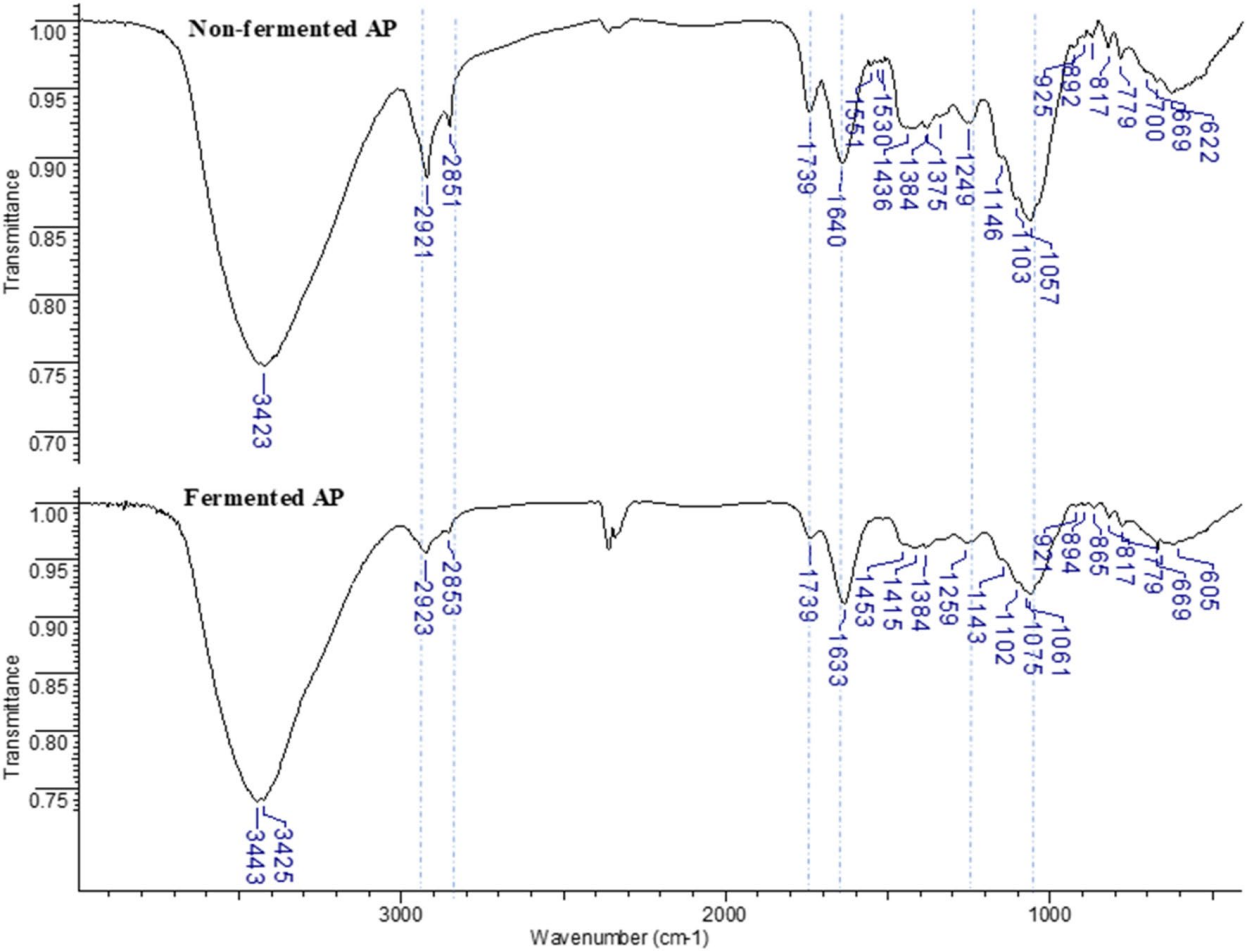
The FT-IR spectrum of non-fermented and fermented AP registered in the spectral range 600–4000 cm^-1^ in the form of KBr pellets. The corresponding bands which differ in the location or intensity in the spectra of non-fermented and fermented AP are marked with a dashed line.

Broad intense bands located in the region of 3440–3420 cm^−1^ derives from the vibrations of –O–H and –N–H functional groups present in e.g. simple and complex carbohydrates, amino acids, carboxylic acid, lignocellulose^56,57^. These bands are of similar intensity and located at comparable wavenumbers both in the FT-IR spectra of non-fermented and fermented AP (Figure 9). Bands at ~2920 and ~2850 cm^-1^ relates to the asymmetric and symmetric stretching modes of –C–H–, –C–H_2_–, –C–H_3_ mainly of carbohydrates. Their intensity is visibly lower in the spectrum of fermented AP compared to the spectrum of non-fermented AP. This is probably due to a decrease in the carbohydrates content due to their contribution to the fermentation process. Band assigned to the stretching vibrations of C=O group in the protonated carboxylic acids is located at 1739 cm^−1^ with lower intensity in the spectrum of fermented compared to the non-fermented AP. The band at 1640 cm^−1^ in the spectrum of non-fermented AP—assigned to the stretching vibrations of C=O in lignin and amide I—was red shift to 1634 cm^−1^ in the fermented AP due to removal of electron-attracting molecules during fermentation process^58^. In the region of 1460–1350 cm^−1^ there are bands assigned to the scissoring vibration of –CH_2_− and in-plane rocking and bending vibrations of –CH_3_. The band at 1249 cm^−1^ in the spectra of non-fermented AP is attributed to the stretching vibration of C–O in phenols^59^. This band is of similar intensity in the spectrum of fermented AP, but is moved to the higher wavenumber. Probably due to increase in the content of non-bonded phenolic acids after the fermentation process. In the range of ~1150–900 cm^−1^ there are bands derived from the stretching vibrations C–O, C–O–C of glycoside bonds, polysaccharides or combinations of sugars with phenolic acids, lignin^60^. In the FT-IR spectrum of fermented AP the relative decrease in the intensity of these bands is observed. There is no distinct differences in the location and intensity of the bands located in the region 100-600 cm^-1^ and assigned to the deformations of the aromatic –C–H.

## Conclusions

The fermentation process led to a change in the phenolic profile of apple pomace. Inoculation with the strain KKP 1527 (*Lpb. plantarum*) allowed, to the greatest extent, to obtain the expected results compared to the other fermentation variants used. It contributed to both the increase in the content of phenolic compounds (TPC) and the value of antioxidant activity. Given the above, it can be assumed that the use of this strain for the fermentation of waste from fruit and vegetable processing, such as apple pomace, may be a promising approach to their reuse for the effective recovery of bioactive substances, in particular gallic acid, chlorogenic acid or caffeic acid. This approach is consistent with the idea of sustainable development due to its ecological nature and process costs compared to conventional methods of artificial synthesis or less efficient independent extraction.

## Data Availability

The data presented in this study are available on request from the corresponding author.
